# Improving healthcare services for living kidney donors: an experience-based approach in the Netherlands

**DOI:** 10.3389/fpubh.2025.1512852

**Published:** 2025-02-25

**Authors:** Wim S. Sipma, Marco van Londen, Annemieke Visser, Margriet F. C. de Jong, Kees C. T. B. Ahaus

**Affiliations:** ^1^Department of Health Services Management & Organisation, Erasmus School of Health Policy & Management, Erasmus University Rotterdam, Rotterdam, Netherlands; ^2^Department of Internal Medicine, Division of Nephrology, University Medical Center Groningen, Groningen, Netherlands; ^3^Department of Applied Health Research (TGO), University Medical Center Groningen, Groningen, Netherlands

**Keywords:** co-design, patient engagement, EBCD, living kidney donation, kidney transplant, patient experience

## Abstract

Living kidney donors voluntarily donate one of their kidneys to someone suffering from end-stage kidney disease. Transplantation is a life-saving opportunity for these patients and generally provides an increase in quality of life. A major goal of research and practice related to living kidney donation concerns the safety of the donor. In comparison, only limited research has been carried out on donor experiences of the donor journey in the full cycle of care. To gain further insight into this, we have performed an experience-based co-design (EBCD) study in a major transplant center in the Netherlands. EBCD is a research approach where professionals and patients share experiences with the purpose of improving the healthcare journey. We identified 11 areas for potential improvement: from intake, throughout surgery and hospitalization, to homecare and aftercare. Donors indicated the importance of person-specific information during the admission process, accurate communication with health professionals and attention to recovery after surgery. Nevertheless, no one regretted their donation, and the overall experience was positive. An important finding for professionals is that donors feel like ‘normal’ patients and want to be taken care of even though they are healthy. Just like other patients, they can be anxious before surgery and some may have concerns about recovery. Although this is a single-center study, the results should be relevant for all transplant centers that are interested in improving donor experiences. We concluded that the EBCD approach, when embedded in a local context, is a valuable tool for bringing patients’ experiences to healthcare improvement.

## Introduction

End-stage kidney disease (ESKD) is life-threatening and requires lifesaving treatment in the form of either dialysis or a kidney transplant. Today, kidney transplantation is the best medical intervention for most ESKD patients, offering a longer life expectancy and an increased quality of life at a much lower cost than dialysis (1–4). Kidney transplants can either be performed with a deceased donor or with a living kidney donor. Globally, in 2021, around 95,000 kidney transplants were performed, of which 35,000 were from living donors (5). Living kidney donation (LKD) is geographically widespread and is performed in around 90 countries. We observed that encouraging LKD has been successful and the number of LKDs now exceeds the number of deceased kidney donations in countries that lack an active deceased kidney donor program (6). Living kidney donors voluntarily undergo surgery and donate one of their two kidneys to an ESKD patient. These are of great value to patient care, as their donations save many lives. Consequently, the care of living kidney donors is of utmost importance (7, 8).

To date, many studies regarding LKD have focused on issues such as the medical suitability of the donor, donor safety, donor motivation, and psychological fitness (1, 9–15). However less research has been done on the donors’ personal experiences of the complete donor procedure. Donor stories have been well documented by donors themselves to inform others, but not with the aim of improving donor care (16–19). The wider literature describes well how patient experiences and patient involvement can be used to improve care pathways (19–24). Therefore, in this study, we collected donor experiences to explore the possibilities to improve the donor care pathway. Our study was inspired by the well-documented experience-based co-design (EBCD) approach that brings both patient ‘user’ experience and healthcare professional experience to healthcare improvement (25, 26). EBCD has its roots in the United Kingdom and is applied in a variety of settings to improve healthcare and patient experiences (24, 27–29). EBCD is a time and resource intensive approach to quality improvement and EBCD projects are customized according to the context, and conducted and reported in different ways (24, 30, 31). However, the core remains: to bring patients’ experiences to healthcare improvement.

This study has three research questions: (a) how do donors experience the current donor care pathway?; (b) where in the care pathway do donors and healthcare professionals see room for improvement?; and (c) which of these improvements should be prioritized in the views of donors and care providers? Through this study we aim to enhance the awareness in the transplant community that donors have personal experiences that go beyond the medical risk analysis of donorship and that these experiences can be used to improve the service provided to the vital group of living kidney donors.

## Methods

### Setting

This single-center study was situated in the University Medical Center Groningen (UMCG) in the Netherlands. The UMCG is a major academic hospital where all types of transplants are performed including kidney, liver, lung, heart, and stem cells. In the UMCG, a multidisciplinary kidney transplant team consisting of nephrologists, surgeons, specialized nursing professionals, and a variety of supporting staff members coordinates all kidney transplants. To date, the UMCG has performed over 5,000 kidney transplants and, over time, developed an active LKD program. As a result, more than 50% of the annual transplants involve living kidney donors. Using the EBCD approach, this study sought to identify areas for improvement in the care process of the LKD program.

### The EBCD approach

The main characteristic of EBCD is that patients and professionals work together to improve a given care pathway. A detailed description of the EBCD approach can be found in the literature (25, 26). Using EBCD, areas of improvement can be found in the moments when professionals and living kidney donors interact. When such interactions have a high impact on the experience of donors they are also referred to as ‘moments of truth’. As far back as 1990, the importance of managing these moments of truth in service delivery was extensively described by Christian Grönroos (32).

The full cycle of the EBCD approach consists of six stages: (a) setting up the project, (b) data gathering from professionals, (c) data gathering from patients/donors, (d) discussing the results with participants and an initial co-design of an improved care pathway, (e) organizing teams for detailed co-design, and (f) a celebration event. Given the complexity of the approach and the significant burden on participants’ time, it is not uncommon to adjust the approach to achieve a better fit with a given context (31, 33–35). Consequently, we customized EBCD to the UMCG context and focused on the first four stages to answer our three research questions.

We started with the first stage, setting up the project, which included developing inclusion criteria for participants. The study was approved by the Medical Ethical committee (METc) of the UMCG (2022/173). All participants were given written information about the study prior to the interviews and signed an informed consent form.

In the second stage, data gathering from professionals, we interviewed nine members of the transplant team (including nephrologists, living kidney donation coordinators, nurse specialists, physician assistant, and social workers). We interviewed them face-to-face, five individually and four in pairs of two. The interviews were summarized by the interviewer and approved in writing by the interviewees. We used a discovery interview technique, aiming to produce a topic list to be used as an interview guide in the face-to-face interviews with the donors (20, 36). After we developed the topic list (Table 1) we started the third stage.

**Table 1:** Topic list for interview with donors.

Introduction and gaining a mutual acquaintance; Explanation of research design
The inside story Reason, what happened and how did it go. From registration to donation and aftercare (if applicable). Exciting moments, uncertainties, joys and/or disappointments. Contacts with UMCG. Experiences throughout the entire process. What stuck with the donor in a positive or less positive (negative?) sense. Timeline of donation process and where are we now?
Stocktaking Who were the key people on the UMCG side? What were your experiences with them? Were there gaps in the moments of contact? Or too much? Which ones? Had there ever been any ambiguities? How were they resolved? In hindsight, what was the most stressful thing in the entire process? Were there any predetermined expectations about the process from sign-up to donation? Was the reality different, if so how? What were your experiences with aftercare? What else was expected? Permanent aftercare or ‘full stop’ at some point? Are aftercare payments going smoothly? What costs do donors bear themselves, is that perceived as reasonable? Specific Was dealing with work discussed? Planning at work, even after admission during recovery. Did the donor have any questions about that? Does the donor expect anything from this through UMCG? How is admission to the UMCG experienced? Are other departments involved? (surgery?). Did recovery go as expected? Could everything be arranged at home? How? Looking back, what was disappointing and what wasn’t so bad?
What could be different? Given the experiences, what would donors do differently in terms of donor screening, kidney donation, aftercare, etc. More specifically: what would donors recommend UMCG to do differently? Which topics do donors think deserve more attention? And where can it be ‘taken down a notch’?

We consider the third stage of EBCD, gathering data on donors’ experiences, as the heart of the process because the ultimate goal of EBCD is to bring user experience to the care improvement process. Data can be gathered in different ways, for instance with surveys or by collecting patient narratives (37, 38). Supported by earlier studies, we chose to collect patient narratives as an appropriate way to collect in-depth and detailed insights into the donors’ experiences and to identify areas for potential improvement (21, 24, 30, 31). We selected nine donors who, following our inclusion criteria, had not had any severe medical issues following surgery. Except one participant who donated shortly after the interview due to circumstances. One donor declined to participate but eight were willing to share their personal donor journey with us. Six donors were female, two were men. The donors’ ages varied between 44 and 74 (mean 56 years) and the time elapsed of seven donors since donation varied between 3 weeks and 40 months (mean 17 months). The donors’ lived experiences were captured in personal stories and resulted in detailed insights into how they experienced the entire donation care pathway from intake and screening to inpatient care and aftercare. As researchers, we concluded that inviting additional donors would not produce significant additional information.

In the fourth stage, discussing results, we first analyzed the data from the interviews and focused on identifying areas for improvement. Two authors (MvL, WS) performed a thematic analysis (39, 40) using Atlas.ti (ATLAS.ti Scientific Software Development GmbH, Berlin, Germany) for initial coding and, after discussion, extracted 11 themes from the data as possible areas for improvement (41).

The next step in the fourth stage was to organize two separate sessions, for professionals and for donors, to discuss the results with both groups. All the participants received the analysis in advance and were asked to think about priorities in the themes for improvement. The first session with professionals was face-to-face, the second session with donors was a virtual meeting using Microsoft Teams (Microsoft Corporation, Albuquerque, New Mexico, United States). Notes were taken during both sessions. Three authors (MvL, WS, AV) were present during the session with professionals, two (MvL, WS) in the MS Teams meeting with donors. We concluded that the 11 themes were recognized by both groups. The group discussions also included an initial setting of priority themes that could be useful for organizing the fifth stage, co-designing improvements in the chosen areas.

## Results

In this section, the 11 themes, the possible areas for improvement, are presented in detail. Since this paper focuses on the inputs from donors, the quotes to support the themes are selected from the donor interviews. The 11 themes that emerged are introduced with a short explanation in Table 2.

**Table 2:** Themes/areas of improvement.

Nr.	Themes – areas of improvement	Explanation
1	Communicate carefully at crucial moments	Think about approvals, adjusted planning (postponements for example), hitches, delays, etc. Personal communication: careful and with the right information. Especially focused on essential moments in the entire process for the donor and recipient.
2	Do not overlook the donor’s social network	Donors like recipients are not alone. They live in a context and receive questions from their environment: about the process, the risks, etc. Families may be worried.
3	Let other donors share their stories	Contact with other donors who are further along in the process, who are also in the middle of it, or who have already done everything, is greatly appreciated.
4	Improve coordination between the Nephrology and Surgery wards	Donors who stay on the Nephrology ward do not have very good experiences of this. The feel ‘forgotten’ on rounds, no surgeon at the bedside, do not feel cared for as a patient.
5	Remember: always a personal approach	Donors are generally satisfied. Nevertheless, there are also some poorer experiences throughout the process. Some of them have to do with treatment in the preliminary phase, but also during admission. Such moments are experienced as annoying.
6	Create excellent experiences: give personal service	Donors greatly appreciate the service they receive. Calm explanations, responses to situations or questions. For example, an offer to take pictures of the operation, arranging another room because of fear of contamination. Keep in mind any concerns they may express about the recipient. Donors are not only concerned with themselves, but also with the recipient.
7	Personalize advice on getting back into good shape after surgery	Donors recover differently. The common thread is that, in the long run, things turn out well, but initial recovery (possibly lasting months) after donation there are some limitations.
8	Be explicit about long-term aftercare for donors	It can be somewhat unclear when aftercare ends: when will I be healthy again?, a donor asked.
9	Organize home care after kidney donation	Home care, mapping out the home situation and the first weeks at home after donation is very important for both donor and recipient. Especially in the case of partner donations or donors living alone.
10	Align donor services at the regional and the university hospital	Donors consider a regional hospital or GP to be a good first contact to look at their suitability for donorship. The UMCG has a different view on this. The hospital is concerned that donors will be incorrectly rejected, or that donorship will be discouraged on incorrect grounds. There is a tension here.
11	Organize adequate support from the general practitioner after LKD	Donors have to rely on their GP for all kinds of medical questions. They would appreciate it if their GP was well informed about being a donor.

### Communicate carefully at crucial moments

The interviews revealed that, during the donation process, some moments are experienced as critical by the donors. Such moments include medical approval for donation, setting a date for surgery, and when the surgery is postponed at the last moment due to urgent events in the hospital. During these crucial moments, both donors and their families can be in a heightened mental state than normal. If communication at such moments is not carefully executed, this can cause feelings of frustration, disappointment, or anger. An example was donor B, who stated: “I was already in the hospital and at the last moment the surgery was canceled. Without any consideration, they told me, ‘We’ll see you again in 3 weeks’. I had carefully planned my business activities for this specific date and put a lot of effort into communicating with my suppliers and customers. So, I got very angry at that moment, whereas in a normal situation, I’m seldom or never angry.”

### Do not overlook the donor’s social network

In the donor care process, information on the process and potential risks are strongly directed at the living kidney donor, and sometimes to the recipient of the donated organ. However, in the interviews, donors indicated that their social context, involving family, colleagues, and neighbors, often lacked accurate information. Donors indicated that those in their social network can also be anxious about risks, especially since many people have no idea what living kidney donation encompasses. Donor E: “My children were worried that I, being their mother, would not be in such a good shape after the donation. So, we had an explanatory conversation on this with the donation coordinator just before the surgery. That helped a lot. Looking back, we should have done this earlier.”

### Let other donors share their stories

The majority of our interviewees had a long period, often many years, to get used to the idea of being a living kidney donor. The main reason for this was that the intended recipient’s illness often resulted in a slow decline of the kidney function before a transplant was appropriate. Donors received a lot of information on LKD, including brochures, oral information in the hospital, and links to numerous websites. An additional way of getting information that was mentioned by donors was to have contact with previous donors. We found that some donors had heard or read the stories shared by earlier donors. These contacts were highly appreciated and could be initiated in spontaneous meetings, for example in the waiting area of the hospital. Donor C: “I really appreciated contact with other donors. I also received a small book with donor stories and how they had experienced the kidney donor process.”

### Improve coordination between the nephrology and surgery wards

Donors are usually hospitalized in the surgical ward of the UMCG, where living donors are admitted both before and after the nephrectomy procedure. Due to COVID-related restrictions and personal preferences, for instance when a parent donates a kidney to a child, some donors were instead hospitalized on the nephrological ward of the UMCG, while still being under care of a surgeon. The nephrological ward, where the transplant recipients are also admitted, is located at a different place in the hospital, a walk of some 500 meters from the surgical ward. A number of donors experienced a lack of attention at this ward due to not being a kidney patient, seeing the ward’s daily routine being organized to take care of kidney patients. A practical example of this was given by donor a: “patients received an information folder during admission. When I asked at the front desk about my information folder it became clear that there were none for donors.

### Maintain a personal approach

Although donors were content with how they were addressed by staff members, in some cases they felt unheard in face-to-face meetings. One example was given by donor C: “One consult with a professional was highly unpleasant. I did not feel respected, I felt vulnerable, and the professional seemed uninterested. I had a nasty feeling afterwards, which was in huge contrast with all my other contacts with the personnel of the UMCG.” Although such experiences were exceptions, nevertheless they are remembered. We noted that donors again and again stressed that the overall experience in the hospital was positive. Although negative personal experiences do occur and are potentially dissatisfiers, the consequence was not a dissatisfied donor.

### Create excellent experiences: give personal service

Elements of personal service are highly appreciated. We found that donor experiences can be positively influenced by letting donors know that exceptions to the routine procedures are possible in case of personal circumstances or personal needs. For instance, donor B mentioned that, on their request, photographs could be taken during the surgery. This was important to the donor as the donor was an active volunteer and wanted to give information on LKD and use the pictures during presentations to share personal experiences as a donor. In another example, donor A stated that, although they were medically ready to be discharged by the hospital, they were allowed to stay a few more days because of personal circumstances: “This made me feel at ease again.”

### Personalize the advice to get in good shape following the surgery

There are differences in the time that donors need to recover, varying from weeks to months, and the issues they experience after the nephrectomy. One donor in our study indicated that they were still not fully being recovered more than a year after the donation. Some donors experience pain, others can be insecure as to what is normal. All have in common that they are admitted to the hospital in good health and leave the hospital in a reduced physical condition. Also, donors differ on their need for assistance to speed up the recovery process. Donor E said “I arrived in the hospital in perfect condition, when I left, I was in a poor condition. I would have preferred to get some help or be given suggestions on how to regain my good condition.” However, after a while, some felt insecure about which daily activities they could safely undertake again, such as running or working in the garden.

### Be explicit about the long-term aftercare for donors

The transplant center offers different forms of medical follow-up once the donor has been discharged from the hospital. Follow-up includes checking on the donor by phone and, after some weeks, a medical check including an iothalamate-measured glomerular filtration rate (mGFR) for testing the kidney function (42). These tests are repeated after 5 and 10 years, as a service to donors who appreciate medical checks. Donors are also advised, in line with international living kidney donor guidelines, to undergo annual health checks, either in the hospital or by visiting their general practioner (GP) (43–45). Donors differed in their wishes regarding follow-up care. Donor F said: “Couldn’t we just visit a GP for check-ups, and should not we end the follow-ups when we are feeling okay?,” while donor A stated “I prefer check-ups in the UMCG, because they have all my medical records and they know me well.” We observed that, after a time, the surgery and the donation move into the background in the lives of the donors. In other words: life goes on. Shared decision-making could be important for donors in making the correct informed decisions on long-term check-ups.

### Organize home care after kidney donations

Especially for donor-recipient couples and for donors who live alone it is important that they receive appropriate home care after the donation. Although the hospital tries to arrange seamless care with external homecare suppliers, this can go wrong. Donor G experienced this: “Home care was requested by the hospital, and I really needed it, I could not lift anything, not even a pan with a meal. However, although a six-week period of supportive home care was requested, I did not get any help at all.” The absence of home care makes daily-life immediately after arriving back home very difficult for donors and donor-recipient couples. Another donor did receive home care and highly appreciated this. Although home care providers are external organizations and it may be difficult to ensure a commitment, to donors it would be very helpful if the UMCG could be given priority when requesting home care.

### Align donor services at the regional and the university hospital levels

In the Netherlands, transplant centers have full responsibility for the donor screening process and for all the steps involved, including mental and physical eligibility. However, donors might prefer an initial screening in a regional hospital closer to home. We observed a tension between the specialists’ desire for a highly professional matching process in the academic setting and the donor-service approach involving some regional prior testing that would be more convenient for the donor. Specialists were concerned that mismatched blood types between donor and recipient might cause a potential donor to be rejected by a regional hospital, whereas they knew that this incompatibility could in some cases be overcome with special treatment. Donor G explained the benefits of an initial regional screening, “I really appreciated that the first tests were undertaken in my own hospital. After that I was referred to the transplant center.” The upside of regional screening for the UMCG could be that potential donors who are clearly not suitable, for instance for sound medical reasons, are not unnecessarily referred to the UMCG. Therefore, aligning the services between regional hospitals and the UMCG could be beneficial for both hospitals and potential donors.

### Organize adequate support from general practitioners after LKD

The healthcare system in the Netherlands is characterized by a prominent role being given to the general practitioner (GP). Some donors indicated that they did not experience adequate support from their GP when they experienced health issues after the LKD. Although GPs are informed about any kidney donations by patients registered at the GP practice, they do not always know how to react to medical concerns by kidney donors. For example, donor A was unnecessarily referred to a nephrologist in the transplant center by his GP. “In the consulting room the nephrologist was clearly surprised by the referral because my complaints were not related to the donation and I was asked ‘Why are you in our hospital?’, and I replied ‘My GP sent me.’.” Given the diversity among GPs, it may be difficult to organize adequate support and to develop the specialized knowledge needed, however donors would appreciate having a well-informed GP.

### Appreciation

In addition, by listening to the donors’ stories and experiences, the interviews also resulted in many compliments from the donors toward the staff members involved in the entire cycle of kidney donation. All the donors were content with the way they were informed about the donation process and with the personal contacts with staff members during all stages of the donation procedure. Also, the easy accessibility of members of the transplant team, the practical information, and the specific information for getting financial compensation that was given to those who were employed or who were independent entrepreneurs were highly appreciated. Also, donors did experience that it was only a small step to make a phone call to the UMCG for some advice after returning home. Furthermore, all donors stated that they would donate again if necessary and no one regretted the decision to become a donor. We noticed that it also became clear that donors themselves can benefit from LKD as illustrated by donor G who stated “Now we can enjoy our lives together again.”

### Priorities

To gain an initial impression of which areas of potential improvement matter most, we also discussed priorities with donors. We found that priorities were determined by personal circumstances. For instance, for donor-recipient couples it was for home care. As a result, the ranking of priorities varied and at first all areas for improvement seemed equally important. Nevertheless, we concluded that careful communication at crucial moments, a personal approach, and advice on how to recover after surgery warranted the highest priority for donors because of the impact of these aspects on the overall experience. In addition, the professionals interviewed indicated that some suggested improvements would be complex to realize, such as organizing home care and support for the general practitioner because these health providers are external, independent organizations beyond the direct influence of the transplant center.

## Discussion

Kidney transplants are of great value in the treatment of end-stage kidney disease (ESKD), and live kidney donation (LKD) is recognized by the World Health Organization (WHO) as an opportunity to reduce the shortage in organ donations (46). In modern healthcare, patient experiences matter. Here, bringing doctor’s and patient’s knowledge together, and viewing patients’ personal experiences as a special kind of knowledge, may lead to better care experiences by patients (25). Although this approach to practicing medicine is of growing importance, in the field of living kidney donation, the donor’s safety dominates (7, 44) and less attention is paid to donors’ experiences. To fill this gap we performed a customized EBCD study to identify aspects that would help improve donors’ experiences during the entire donation journey. To the best of our knowledge, this is the first EBCD study in the field of LKD. By interviewing and discussing their experiences with nine transplant professionals and eight living kidney donors we were able to identify 11 areas for improvement. The personal stories showed a wide variety of experiences, both positive and negative, ideas for improvement, and personal benefits regarding kidney donation.

Our first research question was ‘how do donors experience the donor care pathway?’. Overall, donors were very satisfied with the donation experience. It also became clear that donors were very motivated to help those close to them and, as reported in earlier research, experience personal benefits from donating a kidney (47). Further, none of our sample regretted the decision to donate and all said ‘they would do it again’, which is consistent with earlier research (15, 47). The stories told by the donors not only revealed areas that could be improved, but also deeper motivations and experiences that offer providers insights on what matters to donors in the donation process. To summarize these experiences in one phrase we could say that ‘donors need to feel they are being taken care of’. This reflects that donors can be just like normal patients: some are anxious when visiting the hospital, nervous before surgery, need security and reassurance after surgery, and can be unsure what will happen after leaving hospital (48). Indeed, at some points, they need a little more personal attention because they are healthy and not sick. Maybe a good comparison that occurred to us is the extra personal care and attention given to pregnant women and those who just have given birth.

As an answer to our second research question, ‘where in the care pathway do donors and healthcare professionals see room for improvement?’, we found 11 areas where improvements could give donors a better experience. These areas encompass multiple aspects of the kidney donor pathway. Most of the identified themes related to personal encounters and information regarding the donor’s patient journey. Donors indicated the importance of personal information during their admission, good communication with the health professional during crucial moments of the donation process, contact with other donors, and a personalized approach. Also, themes regarding recovery after surgery, the organization of home care after leaving hospital, the role of regional non-academic hospitals, and adequate briefing of the general practitioner were important. The themes indicated clear and often key moments in the donor care pathway that could be improved. We found support for these issues when discussing them with the professionals. Overall, we concluded that donors, even though they are very healthy, should be considered as ‘normal’ patients with similar needs, anxieties, and expectations.

Our third research question was ‘which of these improvements should be prioritized in the views of donors and care providers?’. Here, careful communication, a personal approach, and recovery after surgery are the improvement areas that would have the greatest impact on the donation experience. Nevertheless, all the themes should be seen as important, and donors’ priorities vary with their personal context and needs. We acknowledge that there will be barriers to achieving improvements. We found that some improvements will be difficult to realize because of the complexity of the healthcare system. For instance, transplant centers and the primary care system do not yet offer integrated post-donation care, and this may be a long-term challenge (8). The same is true for organizing home care. We concluded that, for an individual improvement, the impact on the donor and its feasibility are two dimensions that need to be considered in setting an implementation agenda. We believe that such an agenda might help to determine which areas should be chosen for detailed co-design, which is the next stage of EBCD. We would stress that continuous co-design, although the next logical step, is not an easy task. We encountered many practical difficulties in meeting with the professionals and donors in individual groups, let alone meeting both groups at the same time in the same location. Doctors and donors have jobs and other obligations, many donors live at a distance from the hospital and traveling to a meeting can consume a whole day. Activities require resources and time, both from donors and from professionals. So, although we saw that, during the project, all participants were eager to participate, getting everybody together for group sessions proved a difficult task. Therefore, although we have successfully identified areas of improvement, this is not enough to ensure a successful co-design and implementation stage. A major challenge will be to keep all the participants together during all stages of an EBCD project. Such difficulties have also been addressed in earlier research (31, 49).

Reflecting on the use of EBCD, we believe that, when fully applied, EBCD is a highly time and effort consuming effort and, furthermore, does not consider implementation. Implementation requires organizational change and needs professional guidance, especially in complex organizations like hospitals. This may explain why successful EBCD projects with real change are rarely described in the literature and, as such, the evidence is ambiguous (30, 31, 34). Patients’ stories do give valuable insights into how they experience healthcare services, but this is insufficient to improve these services (49). Nevertheless, this study shows that a customized EBCD approach can identify valuable and practical opportunities for improving patients’ experiences, and that some of these opportunities are relatively easy to implement.

### Strengths and limitations

This study has some limitations that should be taken into account when interpreting the results. First, the study was conducted in a single large transplantation center in the Netherlands. The results of applying this adopted EBCD approach should be seen in this context. However, this also makes EBCD a powerful tool, because of the involvement of local professionals and patients, and several of the identified themes are universal such as ‘communication at crucial moments’. Second, the donors in our study all experienced a rather uneventful donation process without major complications whereas, in reality, a variety of medical complications can occur after kidney donation.

Despite these limitations, we were able to formulate several avenues for improving donor care that are very practical and may be of use in many hospitals across the transplant community. We used EBCD as an established method for improving patient care, and the use of storytelling proved to be an appropriate way to identify areas in the donor care pathway that could be improved (21, 26). The customized EBCD approach was useful in involving donors and professionals and may inspire care givers to broaden their view on how donors experience the donation process. We suggest two areas where future work could be beneficial: first, more research is needed on donor experiences and how to optimize these in different stages of the donor journey; and, second, seeking new ways to customize EBCD approaches such that they are less time and resource consuming but still lead to the ultimate goal: bringing users’ experiences to healthcare improvements.

The main lesson we learned is that really listening to the stories of patients and of donors can generate a lot of inspiring and practical ideas for improving care pathways. A customized version of EBCD would be a good starting point because experiences matter and this approach may bring healthcare to a more patient-engaged level to organize care based on ‘hearing the voice of the people served’ (50, 51).

In conclusion, this study shows that a customized EBCD approach can identify valuable and practical opportunities for improving patients’ experiences, and that some of these opportunities are relatively easy to implement.

## Data Availability

The datasets presented in this article are not readily available because data cannot be shared publicly because of confidentiality. For further information please contact the office of the Medical Ethics Review Board (METc) of the University Medical Center Groningen (UMCG), email metc@umcg.nl. Data are available upon request for researchers who meet the criteria for access to confidential data. Requests to access the datasets should be directed to m.f.c.de.jong@umcg.nl.
